# Precision modeling of mitochondrial disease in rats via DdCBE-mediated mtDNA editing

**DOI:** 10.1038/s41421-021-00325-7

**Published:** 2021-10-19

**Authors:** Xiaolong Qi, Xiaoxu Chen, Jiayin Guo, Xu Zhang, Haifeng Sun, Jianying Wang, Xuezhen Qian, Bo Li, Lei Tan, Lei Yu, Wei Chen, Lianfeng Zhang, Yuanwu Ma, Bin Shen

**Affiliations:** 1 Key Laboratory of Human Disease Comparative Medicine, National Health Commission of China (NHC), Institute of Laboratory Animal Science, Chinese Academy of Medical Sciences, Peking Union Medicine College, Beijing, China; 2 Beijing Engineering Research Center for Experimental Animal Models of Human Critical Diseases, Institute of Laboratory Animal Science, Chinese Academy of Medical Sciences, Peking Union Medicine College, Beijing, China; 3 State Key Laboratory of Reproductive Medicine, Nanjing Medical University, Nanjing, Jiangsu China; 4 Gusu School, Nanjing Medical University, Nanjing, Jiangsu China; 5 Neuroscience Center, Chinese Academy of Medical Sciences, Beijing, China; 6 Center for Global Health, School of Public Health, Nanjing Medical University, Nanjing, Jiangsu China; 7 Women’s Hospital of Nanjing Medical University, Nanjing Maternity and Child Health Care Hospital, Nanjing Medical University, Nanjing, Jiangsu China

**Keywords:** Genomic analysis, Mechanisms of disease

Dear Editor,

Mitochondrial DNA (mtDNA) mutations manifest mainly as base changes, resulting in a broad spectrum of serious diseases in human^1^. To date, more than 270 pathogenic variants of mtDNA in humans have been reported and the number continues to rise^2^. Currently, no curative treatments for patients with mtDNA pathogenic mutations are available. There is an urgent need for the generation of animal models harboring precise human mtDNA variants to reveal the physiopathology and develop therapeutic approaches for these diseases. Mammalian animal models with pathogenetic mtDNA mutations could be generated by mitochondrial transplantation or screening new mtDNA mutations by using *PolG*^D257A/WT^ lineages^3^, whereas neither of these strategies could produce animals with precise mtDNA mutations on demand. Recently, a bacterial toxin deaminase (DddA) from *Burkholderia cenocepacia*, was reported to be able to convert cytosine to uracil specifically within dsDNA. The toxin domain of DddA (DddA_tox_,1264–1427 amino acids) could be engineered and incorporated with mitoTALE system to efficiently achieve C ∙ G-to-T ∙ A conversion in mtDNA of human cell lines^4^. This breakthrough technology, DddA-derived cytosine base editor (DdCBE), paves a new way to produce animal models with desired mtDNA mutations. Lately, DdCBE is used to mediate mtDNA editing in mouse, but does not cause apparent phenotype^5^. Rat is an important and widely used laboratory model and has many advantages, especially in physiology, toxicology, and pharmacology study. In this study, we explored the application of DdCBE in rats to generate a mitochondrial disease model with pathogenetic mtDNA mutations.

In humans, G8363A mutation in the mtDNA *TRNK* gene is associated with myoclonus epilepsy with ragged-red fibers (MERRF) syndrome, cardiomyopathy, and Leigh Syndrome^6^. G14710A mutation in the mtDNA *TRNE* gene is associated with mitochondrial myopathy^7^. To mimic these two mtDNA mutations in rats, G7755, and G14098, corresponding to human G8363 and G14710 respectively, were selected for targeting (Supplementary Fig. S1a, b). DdCBE vectors were assembled by one-step Golden Gate assembly using an RVD library containing 192 modules that we developed previously^8^, resulting in four DdCBE pairs for each site (Left-G1333C (L1333C) + Right-G1333N (R1333N), L1333N + R1333C, L1397C + R1397N, and L1397N + R1397C). First, we transfected rat glioma C6 cells with each DdCBE pair and tested their efficiency. Sanger sequencing results indicated that DdCBE could mediate G-to-A conversion at both G7755 (Supplementary Fig. S1c) and G14098 loci (Supplementary Fig. S1d). Deep sequencing data showed that different DdCBE pairs could mediate variable editing efficiency (Supplementary Fig. S1e, f). For the G7755 site, the L1397N + R1397C pair achieved the highest editing (16.33% ± 1.97%) (Supplementary Fig. S1e), while the L1397C + R1397N yielded the highest editing for G14098 with 19.97% ± 1.35% efficiency (Supplementary Fig. S1f). These results collectively demonstrate that DdCBE pairs can produce efficient base editing at designated mtDNA sites in rat cells.

Then we attempted to achieve in vivo mtDNA editing by injecting mRNAs of DdCBE pair with the best performance to rat zygotes. Among 119 pups born, no editing events were detected at either G7755 or G14098 site, even though the injection concentration of mRNA was high up to 200 ng/μL (Supplementary Table S1). DdCBE-mediated base editing relies on active mtDNA replication, whereas mtDNA replication is inactive in the fertilized egg till the preimplantation stage in mammalian embryos^9^. We reasoned that continuous expression of DdCBE pair could conquer the problem. Therefore, further tests were performed by cloning DdCBE pair into the PiggyBac vector and co-injecting rat 1-cell embryos with transposase mRNA (Supplementary Fig. S2a). As a result, one out of four pups for G7755 was successfully edited with 36.33% efficiency (Fig. 1a, b; Supplementary Table S1). Notably, all pups for G14098 were edited with mutation loads ranging from 3.91% to 46.73% (Fig. 1a, b; Supplementary Table S1). We then amplified DddA_tox_ half sequence to verify the transgenesis of DdCBE. Unexpectedly, only G7755A founder #2, and #1, #2, #6 from G14098A founders were detected with very week signals (Supplementary Fig. S2b, c). This observation indicated that, although DdCBE pair almost failed to integrate into the rat genome by transposon system, the transient expression of DdCBE pair via PiggyBac vector could last longer than that via mRNA injection, and facilitated C ∙ G-to-T ∙ A conversions in mtDNA. It is further proved by microinjection only with DdCBE pair in PiggyBac vector targeting G7755. Out of 26 F0 rats, nine pups were successfully edited at G7755 (Supplementary Fig. S3a and Table S1), and only two pups were detected with DdCBE transgene (Supplementary Fig. S3b). To characterize the editing status in founders, we collected tissues from G14098A #2 and #7 founders for sequencing. The results revealed that mtDNA editing was detected with variable efficiency in different organs (Supplementary Fig. S2d, e). Taken together, prolonged-expression of DdCBE pair via PiggyBac vector facilitates efficiently systemic mtDNA editing, including in the ovary.

**Fig. 1:**
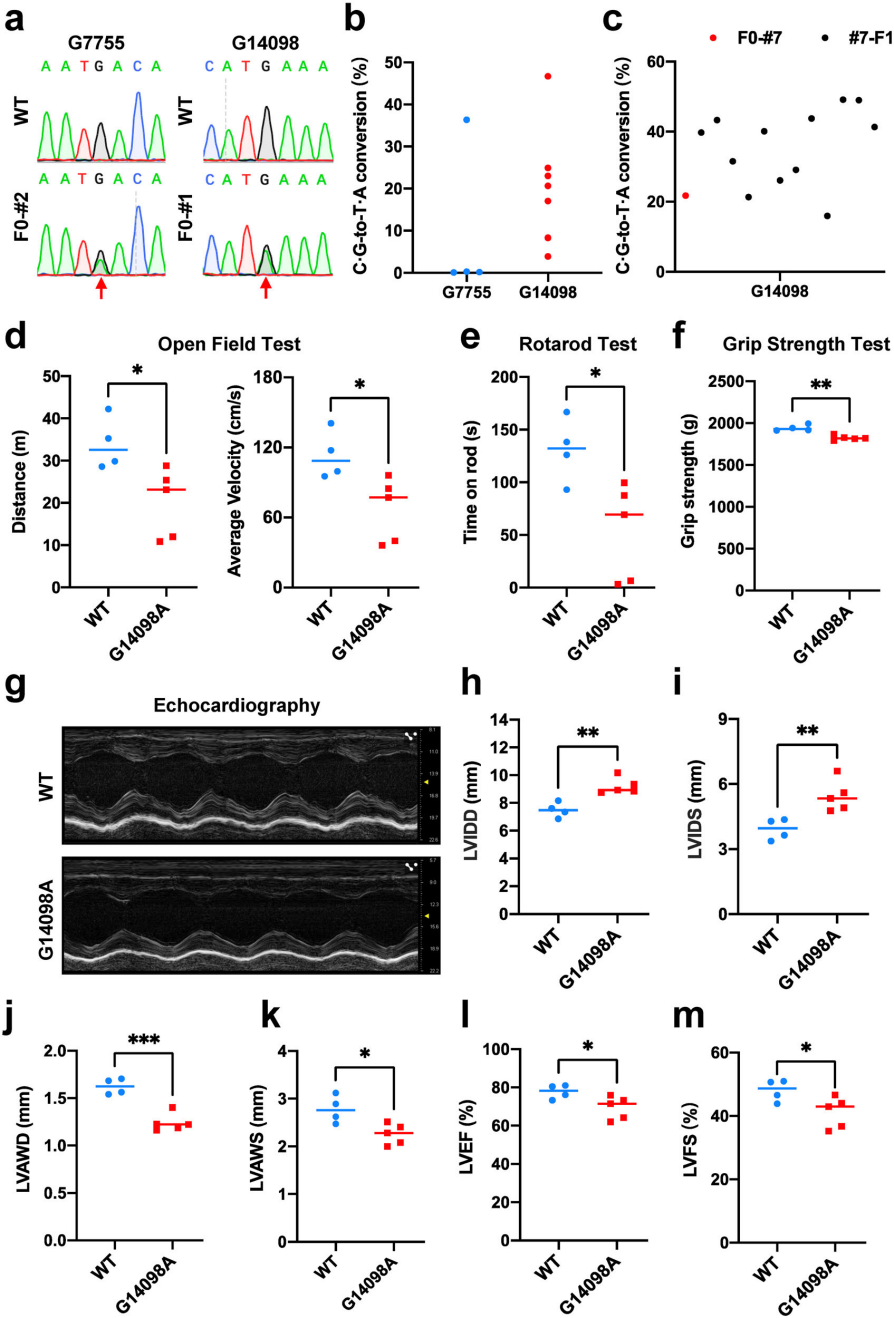
Mitochondrial disorder modeling by DdCBE in rat. **a** Representative sequence chromatograms of G7755A (Left) and G14098A (Right) founder rats. Target sites are indicated by red arrows. **b** Frequencies of G-to-A conversion at G7755 (Blue dots) and G14098 (Red dots) in founder rats. **c** Frequencies of mtDNA G-to-A conversion of offspring from G14098A #7 founder. The red dot indicates founder, black dots indicate F1 offspring. **d** Measuring distance (Left) and speed (Right) by open field test. **e** Evaluation of motor coordination by Rotarod test. **f** Measuring grip strength by Grip Strength Test. **g**–**m** Echocardiography analysis of wild-type rats and edited F1 males. The tests include a snapshot of M-mode of echocardiography (**g**), left ventricular (LV) diameter at end-diastole and end-systole (LVIDD, LVIDS) (**h**, **i**), LV anterior wall thickness at end-diastole and end-systole (LVAWD, LVAWS) (**j**, **k**), LV ejection fraction (LVEF) (**l**), and LV percent fractional shortening (LVFS) (**m**). Data were presented as a scatter dot plot with means (*n* = 4 for wild-type control, *n* = 5 for G14098A F1 rats). Significance was calculated with unpaired two-tailed Student’s *t*-test (*P < 0.05, **P < 0.01, ***P < 0.001).

To investigate whether established G-to-A mtDNA mutation is inheritable, we superovulated female G14098A founder #7 and collected its oocytes for in vitro fertilization (IVF). The fertilized zygotes were transferred into the oviduct of pseudopregnant females to generate F1 offspring, while the unfertilized oocytes were subjected to genotyping. G14098A mutation could be detected in unfertilized oocytes, revealing that DdCBE-mediated editing might be transmittable (Supplementary Fig. S4a). Accordingly, 12 out of 28 F1 pups were detected with G14098A mutation by Sanger sequencing (Supplementary Fig. S4b), demonstrating that DdCBE-mediated mtDNA editing could be transmitted to the next generation. Deep sequencing results further revealed that the mutation loads of 12 F1 offspring ranged from 15.92% to 49.15% (Fig. 1c; Supplementary Table S2). Interestingly, ten offspring harbored higher mutation loads than their mother (Fig. 1c), suggesting that it is quite possible to obtain individuals with higher mutation load in future generations. The presence of DdCBE transgenesis in all 12 edited F1 pups was examined and no positive signal was observed (Supplementary Fig. S4c), which further confirmed that transient expression of DdCBE pairs in founder could achieve heritable mtDNA editing. To characterize the systemic editing status in the F1 generation, we collected various tissues from two G14098A F1 rats (#13 and #25) for sequencing. The results revealed comparable mutation loads in variant tissues of F1 rats (Supplementary Fig. S5a, b).

We next investigated the consequences of introducing pathogenic mtDNA variant in rat. In humans, G14710A mutation, resulting in an anticodon swap (Glu to Lys) in *MT-tRNA*^*Glu*^, was reported to be associated with mitochondrial myopathy^7^. We sacrificed G14098A founder #1, which carried the highest editing efficiency among founders (46.73%) (Supplementary Table S3), and collected tissues to test the ATP level and complex I activity. Compared to the wild-type rat, we detected decreased ATP level (Supplementary Fig. S6a) and complex I activity (Supplementary Fig. S6b) in the heart and brain of founder #1, with no changes in the liver. Encouraged by these results, we further analyzed the motor ability of F1 males with 26.11% to 49.15% editing (Supplementary Table S3) using open field test (OFT), Rotarod Test, Grip Strength Test, Morris Water Maze (MWM) test, and Tail Suspension Test (TST). The OFT results showed that G14098A F1 males exhibited decreased movement distance and average speed compared to the control (Fig. 1d). The motor coordination and balance, and forelimb grip strength of edited F1 rats were also impaired (Fig. 1e, f). However, no difference was detected in the MWM Test and TST, indicating that the learning ability and depressive state were not affected in these edited rats (Supplementary Fig. S6c, d). These data suggested that G14098A mutation in mtDNA could impact the motor ability, but not the learning performance and emotional status. Besides muscle, the heart is also an organ with high energy demands. Mitochondrial dysfunction has been widely observed in the failing heart^10^. Using echocardiography, we assessed the cardiac structure and function of G14098A F1 males at 4 months of age and found that G14098A mutant rats exhibited dilated cardiomyopathy phenotype with larger chambers and thinner walls, and decreased function of contraction (Fig. 1g–m). These changes were demonstrated by increased left ventricular (LV) diameter at end-diastole and end-systole (LVIDD, LVIDS) (Fig. 1h, i), decreased LV anterior wall thickness at end-diastole and end-systole (LVAWD, LVAWS) (Fig. 1j, k), decreased LV ejection fraction (LVEF) (Fig. 1l), and LV percent fractional shortening (LVFS) (Fig. 1m). The decreased ATP level (Supplementary Fig. S7a) and complex I activity (Supplementary Fig. S7b) were also detected in heart of G14098A F1 rats. To further confirm our results, we generated F2 offspring from edited F1 females and selected five unedited males and five edited males with frequencies of 31.95%–43.31% to analyze their motor ability and cardiac function at 7 weeks of age (Supplementary Table S3). Consistent with the results of F1 rats, the edited F2 males also displayed impaired motor ability and cardiac function (Supplementary Fig. S7c–k). Theoretically, the human G14710A mutation interferes with the decoding process of the tRNA and affects translation. Unexpectedly, we didn’t detect obvious changes in proteins encoded by mtDNA in rats (Supplementary Fig. S7l). We speculate that the wild-type *MT-tRNA*^*Glu*^ may have a competitive advantage over the mutant tRNA during the decoding process, and translational defects will not appear until a quite high mutation load is detected. Collectively, these results demonstrate that precise DdCBE editing in rats can be applied to model human mitochondrial disease.

To evaluate the off-target activity of DdCBE in the rat mtDNA, we first analyzed the deep sequencing data to detect the presence of DdCBE-induced off-target editing. In C6 cells, DdCBE pairs selected for injection (L1397N + R1397C for G7755 and L1397C + R1397N for G14098) showed lower than 0.2% undesired editing (Supplementary Fig. S8a, b). Notably, the L1333N + R1333C pair of DdCBE mediated efficiently editing at G7757 (9.84% ± 0.17%) within the spacing region, indicating that C within 5′-aC context could also be converted to T by certain DdCBE pair (Supplementary Fig. S8a). In rat founders, both G7755-DdCBE and G14098-DdCBE showed off-target editing with a frequency below 0.1% (Supplementary Fig. S8c, d). To further profile the off-target activity of DdCBE on the entire mitochondrial genome, we performed whole mtDNA sequencing in founders. The results showed that sparse unwanted editing events with a frequency lower than 2.5% (Supplementary Fig. S9a). We also noticed that most off-target sites (OTS) were concentrated around the target site in G14098A founders (Supplementary Fig. S9a), indicating that these off-target editing may be induced by the unstable binding of the DdCBE pair. The OTS with a conversion rate over 0.3% in any sample were selected for further analysis (ten sites for G7755A founder and 24 sites for G14098A founders) (Supplementary Fig. S9b, c). The frequency of off-target edits were all below 0.8% at ten sites in G7755A founder (Supplementary Fig. S9b). G14098A founder #1, which harbored the highest on-target editing, showed the highest off-target editing rate at 19 sites compared with other founders (Supplementary Fig. S9c), indicating a strong correlation between on-target editing efficiency and off-target activity. Among the 34 OTS in founders, 29 (85.29%) sites were detected within 5′-tC or 5′-tcC motifs, suggesting that the off-target editing indeed arose from DddA_tox_ (Supplementary Fig. S9b, c). Collectively, DdCBE can mediate relatively precise base editing in rat mtDNA with limited off-target editing.

In summary, we showed for the first time that effectively targeted editing of mtDNA by DdCBE in rats resembling human mitochondrial diseases, which provides important in vivo models to study the mechanisms of mitochondrial disorders and is also useful for the development of clinical therapies. Although the limited off-target editing detected in rat mtDNA may not be sufficient to display phenotypes, a more precise DdCBE should be developed in future studies to accurately simulate human mitochondrial diseases in animal models.

## Supplementary information

Supplementary Information

## Data Availability

The high-throughput sequencing data have been deposited to the NCBI Sequence Read Archive database (accession ID, PRJNA725092).
