# Supplementary material for: Precision modeling of mitochondrial disease in rats via DdCBE-mediated mtDNA editing
Source: Cell Discov. 2021 Oct 19;7:95. doi: 10.1038/s41421-021-00325-7 (PMC8523528; doi:10.1038/s41421-021-00325-7)
Supplement: Supplementary file 1 — Supplementary Information [file 41421_2021_325_MOESM1_ESM.pdf]

Supplementary information

Precision modeling of mitochondrial disease in rats via DdCBE-mediated mtDNA editing

Xiaolong Qi[1, 2*], Xiaoxu Chen[3*], Jiayin Guo[3, 4*], Xu Zhang[2*], Haifeng Sun[3*], Jianying Wang[3], Xuezhen Qian[3], Bo Li[1], Lei Tan[3], Lei Yu[2], Wei Chen[2], Lianfeng Zhang[2, 5], Yuanwu Ma[1, 2, 5]†, Bin Shen[3, 4, 6, 7]†

Contents

Materials and Methods

Figure S1. Mitochondrial genome editing by DdCBE in C6 cells.

Figure S2. Mitochondrial genome editing by DdCBE in rats.

Figure S3. Mitochondrial genome editing by injection of pure DdCBE constructions.

Figure S4. Germline transmission analysis of G14098A mutation.

Figure S5. Mitochondrial genome editing in various tissues of G14098A F1 rat.

Figure S6. Analysis of ATP synthesis, complex I activity and motor ability in G14098A F1 rat.

Figure S7. Phenotype analysis of G14098A F1 and F2 rat.

Figure S8. Deep sequencing analysis of DdCBE off-target.

Figure S9. Off-target analysis by whole mtDNA sequencing.

Table S1. DdCBE Injection Summary.

Table S2. Summary of germline transmission of mtDNA mutations.

Table S3. Information of edited rat.

Table S4. Information of primers.

Supplementary sequence

Materials and Methods

Animals

Sprague Dawley rats used in this study were purchased from Beijing Vital River Laboratories Animal Technology Co., Ltd. and housed in an AAALAC accredited animal facility. All animal experiments were approved by the Institutional Animal Care and Use Committee (IACUC) of the Institute of Laboratory Animal Science, Chinese Academy of Medical Sciences & Peking Union Medical College (IACUC-ZLF18003).

DdCBE and PB-DdCBE plasmid construction

RVD library was used to construct DdCBE vectors [1] and can be obtained from Addgene (Shen Lab DdCBE kit). Briefly, RVDs and backbone plasmids were mixed with Bsa I (NEB), T4 DNA ligase (NEB) and reaction buffer in a single tube. Reaction was performed in a thermocycler with following program: 10 cycles of 37°C for 10 min and 16°C for 10 min; 50°C for 5 min; 80°C for 5 min. The assembled plasmids were chemically transformed into *Escherichia coli* DH5α competent cells (Transgene) and confirmed using PCR and Sanger sequencing. DdCBE was cloned into PiggyBac (PB) vector (PiggyBac Transposon Vector System, GENTAUR) to construct PB-DdCBE. Sequences of primers and DdCBE are listed in Table S4 and Supplementary sequence.

Cell culture and nucleofection

The rat C6 cells were cultured in DMEM supplemented with 10% FBS (Gemini), 100 units/mL penicillin and 100 μg/mL streptomycin at 37°C with 5% CO_2. For C6 cell nucleofection, 400 ng of left and right DdCBE each were mixed in the nucleofection reagent. Then 10^5 C6 cells were nucleofected with premixed DdCBE vectors using Lonza 4D-Nucleofector. Cells were cultured with fresh medium with 0.6 μg/mL

puromycin 24 hrs post transfection and collected at day 3 for DNA extraction.

In vitro transcription of DdCBE mRNA

DdCBE plasmids were linearized with Pme I. The linearized plasmids were purified and used as template for *in vitro* transcription using mMESSAGE mMACHINE T7 Ultra Kit (Life Technologies) according to the manufacturer's manual. DdCBE mRNA was purified with RNA Clean & Concentrator™-25 (Zymo Research) and stored at -80°C until use.

Microinjection of rat fertilized eggs

Microinjection of fertilized rat eggs was described as previously reported [2]. In brief, four- to six-week-old female rats were i.p. injected with 30 units of pregnant mare serum gonadotropin (PMSG, Sigma-Aldrich), followed by an i.p. injection of 30 units of human chorionic gonadotropin (hCG, Sigma-Aldrich) 48 hrs later, and immediately mated with male rats. On the next day morning, the eggs were collected from ovarian ducts of superovulated female rats and cultured in KSOM (Millipore) at 37°C, 5% CO_2. DdCBE pair's mRNA (30, 100 or 200 ng/μL) was injected into the cytoplasm of zygotes using a Nikon microinjection system. 10 ng/μL of PB-DdCBE and 30 ng/μL of Super PiggyBac transposase mRNA were injected into the pronucleus of zygotes. The injected zygotes were transferred to pseudopregnant SD rats (20-30 zygotes per pseudopregnant SD rat).

Germline transmission detection

Edited female rats at the age of five-week-old were i.p. injected with PMSG and hCG for superovulation. Then oocytes were collected for *in vitro* fertilization and further

transferred to pseudopregnant SD rats for pups born.

DNA extraction and genotyping

Genomic DNA of C6 cells was extracted with QuickExtract™ DNA Extraction Solution (Lucigen). Genomic DNA of rat tissues was obtained by proteinase K digestion and phenol-chloroform extraction. The amplicon spanning targeted region was amplified and subjected to Sanger sequencing. Primers are listed in the Table S4.

Open Field Test (OFT)

OFT was conducted in an 80 cm × 80 cm × 50 cm black box. The behavior was recorded for 5 min by SuperMaze digital tracking system. All tracks were analyzed by SuperMaze software.

Rotarod Test

To assess the motor behavior in rats, a rotarod machine with automatic timers and falling sensors (ZH-600B, Anhui Zhenghua Biologic Apparatus Facilities) was used. In brief, all rats were habituated to stay on the stationary drum for 3 min before the training sessions. Habituation was repeated every day before the session. The rat was placed back on the drum immediately after falling, up to 5 times in one session, at a relatively slow speed (20 rpm). The test was repeated one session a day for three consecutive days. The latency to falling was recorded automatically by photo-cells.

Grip Strength Test

To evaluate the muscular strength, grip strength was recorded as the average of three consecutive trials for limbs, using a Grip Strength Meter (ZH-YLS-13A, Anhui Zhenghua Biologic Apparatus Facilities).

Morris Water Maze (MWM) test

MWM test was conducted in a round black tank with 150 cm in diameter and 60 cm in depth. The water temperature was maintained at 25 ± 0.5°C. The escape platform, a 12 cm Plexiglass circle in diameter, was placed in the center of one quadrant of the tank and submerged 1 cm beneath the water surface. The platform remained in the same position throughout the learning trials and visual cue tests, and then removed from the tank during the probe test. All rats were trained in two trails per day with an inter-trial interval of 30 min. The spatial reference memory was evaluated after five consecutive days of training. The trajectory was recorded using a SuperMaze digital tracking system.

Tail Suspension Test (TST)

The rat tail was fixed and suspended to a horizontal bar for 6 min. The head was 15 cm away from the table. The immobility time of the rats was recorded by SuperMaze digital tracking system during the last four minutes (3-6 min).

ATP measurement

The ATP level of brain, liver and heart tissues was detected using ATP Colorimetric/Fluorometric Assay Kit (K354-100, BioVision, Inc.). Briefly, 10 mg fresh tissue was homogenized in 100 μL ATP Assay Buffer, followed by deproteinization using ReadiUse™ TCA Deproteinization Sample Preparation Kit (19501, AAT Bioquest, Inc.). 30 μL prepared sample and ATP standards were added to 96-well plate and the volume was adjusted to 50 μL/well using ATP assay buffer. Then 50 μL reaction mix (44 μL ATP assay buffer, 2 μL ATP probe, 2 μL ATP converter and 2 μL developer) was added to each well. After incubation at room temperature for 30 min, a micro-plate

reader (Multiskan FC with Incubator 51119100, Thermo) was used to measure the light absorption value (OD 570 nm). The ATP level was calculated based on the standard curve.

Complex I Enzyme Activity Assay

The complex I enzyme activity of brain, liver and heart tissues was detected by using Complex I Enzyme Activity Assay Kit (ab109721, Abcam). Briefly, 200 mg fresh tissue was homogenized in 1 mL cold PBS using a Dounce, followed by adjusting concentration of the sample with PBS to 5.5 mg/mL. Then the protein was extracted from the sample by adding 1/10 volume of detergent solution and was placed on ice for 30 min. The supernatant was collected and transferred to a clean tube after 16,000 g centrifuge for 20 min at 4°C. Then 200 μL of samples, positive control and buffer only control were loaded to the well. After incubation for 3 hrs at room temperature and wash three times with 300 μL 1× wash buffer, 200 μL of assay solution (contain NADH and Dye) was added to each well to detect absorption value (OD 450 nm) using a micro-plate reader. The activity was presented as the changes in absorbance per minute per amount of sample loaded into the well.

Echocardiography analysis

The heart function was evaluated by echocardiography analysis as described before [2]. Briefly, the rat was anesthetized with isoflurane and echocardiographic observation was performed using a micro-ultrasound imaging system (Vevo 3100). Measurements were recorded at least three continuous cardiac cycles.

Western blotting

The heart tissues were lysated with RIPA buffer supplemented with protease inhibitor cocktail (Bimake) for protein extraction. Proteins were separated by 10% SDS/PAGE gel following the standard procedures. Primary antibodies anti-ND2 (Proteintech 19704-1-AP; 1:600), anti-MTCO1 (Invitrogen 459600; 1:2,000), anti-MTCO2 (Proteintech 55070-1-AP; 1:2,000), anti-MTCO3 (Proteintech 55082-1-AP; 1:500) and anti-GAPDH (Beyotime AF0001; 1:3,000) were used. HRP-conjugated secondary antibodies (Bioworld) were diluted at a concentration of 1:5,000. Protein signals were measured using enhanced ECL Western blot Substrate (Vazyme) and visualized with Tanon imaging system.

Deep sequencing

Genomic regions of interest were firstly amplified with barcoded primers (first round PCR, PCR1) using Phanta Max Super-Fidelity DNA Polymerase (Vazyme). The PCR1 products were pooled with equal moles and purified for the second round PCR (PCR2). PCR2 was performed using index primers (Vazyme) and purified by DNA Clean Beads for sequencing using Illumina NovaSeq platform. Barcoded primers used for PCR1 are listed in Table S4.

Whole mtDNA sequencing

Long-range PCR was used to capture whole mtDNA as previously reported [3]. In brief, two overlapping fragments (F1 and F2) around 8 kb each were amplified with Phanta Max Super-Fidelity DNA Polymerase (Vazyme). The two fragments were purified by Gel extraction, pooled with equal moles and subjected to libraries preparation using TruePrepTM DNA Library Prep Kit V2 for Illumina (Vazyme). The libraries were

purified using DNA Clean Beads by 0.5x/0.35x double size selection. Libraries were and sequenced by Illumina NovaSeq platform. Primers for amplification of long-range PCR are listed in Table S4.

Deep sequencing data analysis

The Rattus norvegicus mitochondrial genome reference sequence (NC_001665) was downloaded from NCBI database. Bowtie2 was used to build the alignment index using default parameters. Paired end reads with overlap were merged into a single read, and bowtie2 was used for alignment in single end mode. Otherwise, reads were mapped in paired end mode by using bowtie2 with default parameters. Alignment results were converted to bam format using samtools and visualized in Integrative Genomics Viewer (IGV). Bases with depth over 2 million were truncated to 2 million, and only C-to-T or G-to-A conversion was calculated for DdCBE-mediated editing.

Whole mtDNA sequencing data analysis

Quality control was performed for the sequencing data by fastqc and trim_galore in paired end mode. The Illumina adapter sequence or Ns in either side of the read was trimmed, and only reads with quality over 20 were kept for further analysis. QC-passed reads were mapped to NC_001665 by using bowtie2 with default parameters of paired end. The DdCBE editing efficiency was calculated as mentioned above.

Off-target analysis

SNP sites of Rattus norvegicus were obtained from Variation VCF in Ensembl database, which includes 19 annotated C·G to T·A variation. For the off-target analysis, only the C·G sites were kept and the following sites were excluded before analysis and

visualization: (1) the above obtained SNP sites; (2) the evident SNP sites of which C/G to T/A variation over 90% in any sample; (3) sites within the DdCBE spacing region.

References

1 Guo, J. *et al*. Precision modeling of mitochondrial diseases in zebrafish via DdCBE-mediated mtDNA base editing. *Cell Disc* (2021). Accepted.

2 Ma, Y. *et al*. Generating rats with conditional alleles using CRISPR/Cas9. *Cell Res* **24**, 122-125 (2014).

3 Mok, B. Y. *et al*. A bacterial cytidine deaminase toxin enables CRISPR-free mitochondrial base editing. *Nature* **583**, 631-637 (2020).

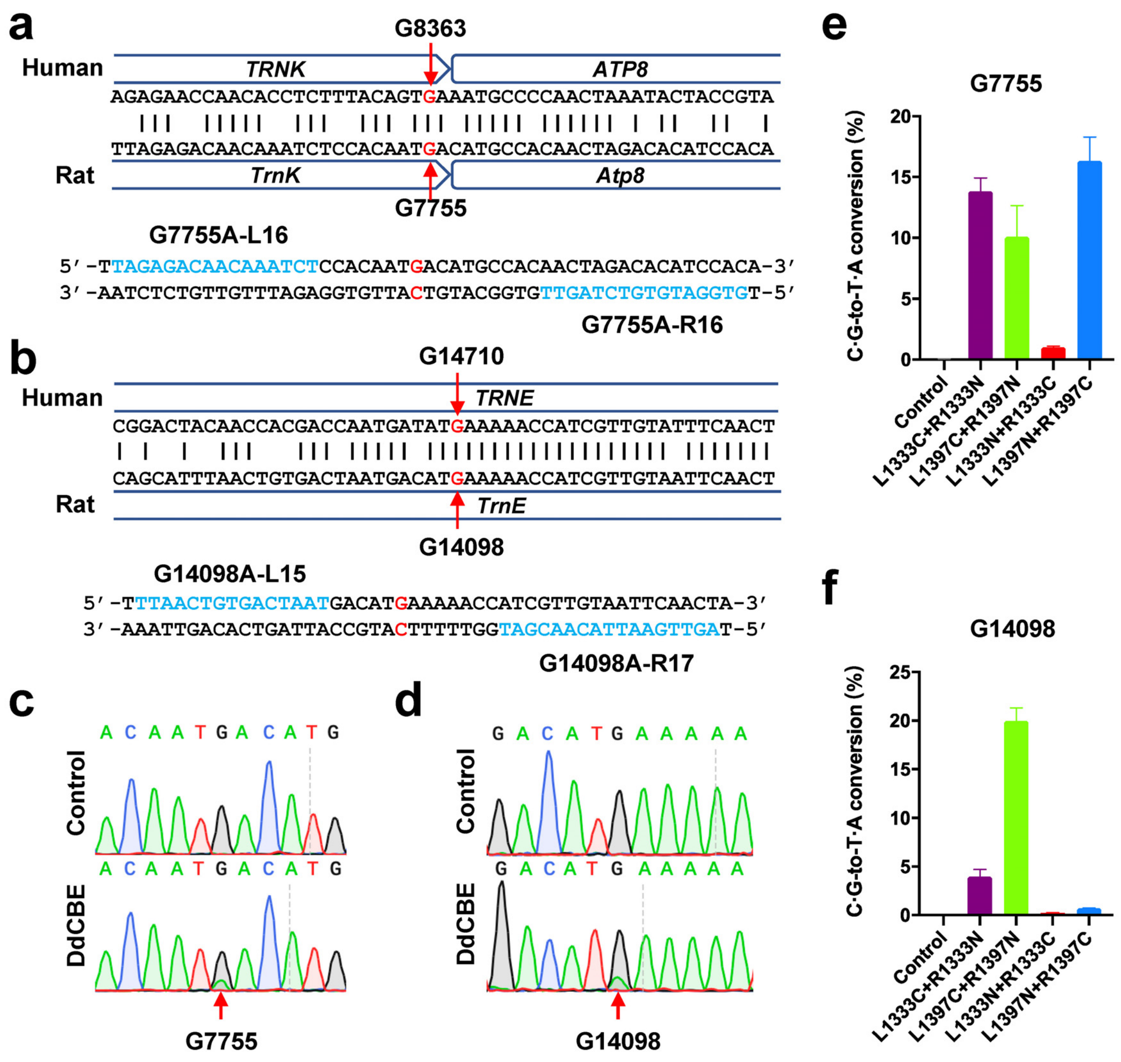

Figure S1. Mitochondrial genome editing by DdCBE in C6 cells.

a, **b** Conservation analysis of G8363 (**a**) and G14710 (**b**) sites between human and rat. The target base is shown in red and indicated by arrow. RVDs recognition sequences are in blue.

c, **d** Sanger sequencing chromatograms of G-to-A conversion in C6 cells transfected with L1397N + R1397C targeting G7755 (**c**) and L1397C + R1397N targeting G14098 (**d**), respectively. Target sites are indicated by red arrows.

e, **f** Frequencies of G-to-A conversion at G7755 (**e**) and G14098 (**f**) in C6 cells treated with different DdCBE pairs.

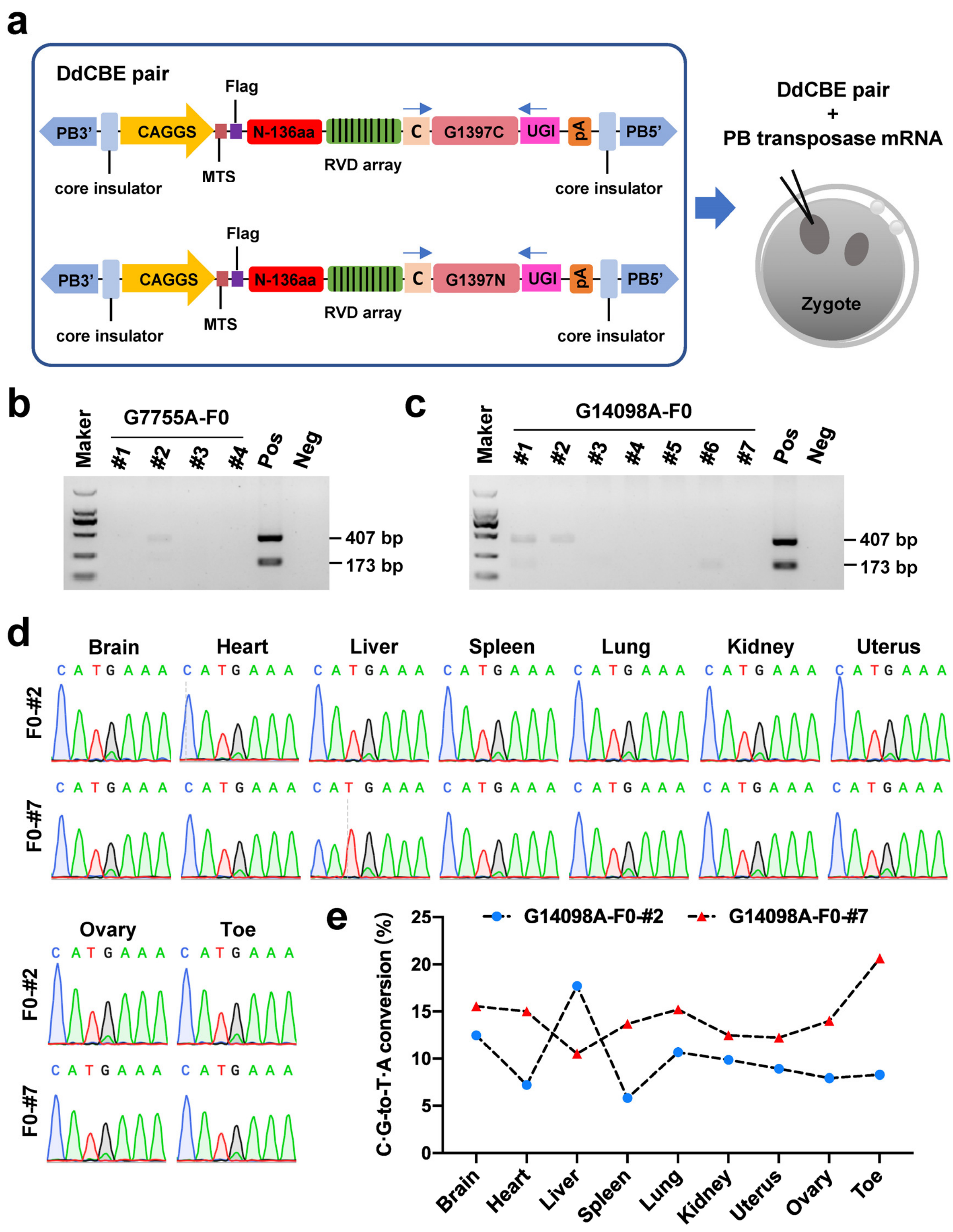

Figure S2. Mitochondrial genome editing by DdCBE in rats.

a PigggBac vectors containing DdCBE pair were co-injected with PiggyBac transposase mRNA into zygotes. Blue arrows indicate primers for amplifying $\mathrm{DddA_{tox}}$ halves.

b, **c** Detecting the presence of $\mathrm{DddA_{tox}}$ halves in the genome of G7755A (**b**) and G14098A (**c**) founders. G1397N and G1397C $\mathrm{DddA_{tox}}$ halves yield a 407 bp and a 173 bp band, respectively. Genomic DNA (gDNA) of DdCBE knock-in rat, which was generated by integrating DdCBE pair into *Rosa26* locus via CRISPR/Cas9-

assisted homologous recombination, was used as a positive control (Pos). Wild type gDNA was used as a negative control (Neg).

d Sanger sequencing chromatograms of G-to-A conversion in tissues of G14098A #2 and #7 founders.

e Deep sequencing analysis of G-to-A conversion in tissues of G14098A #2 and #7 founders.

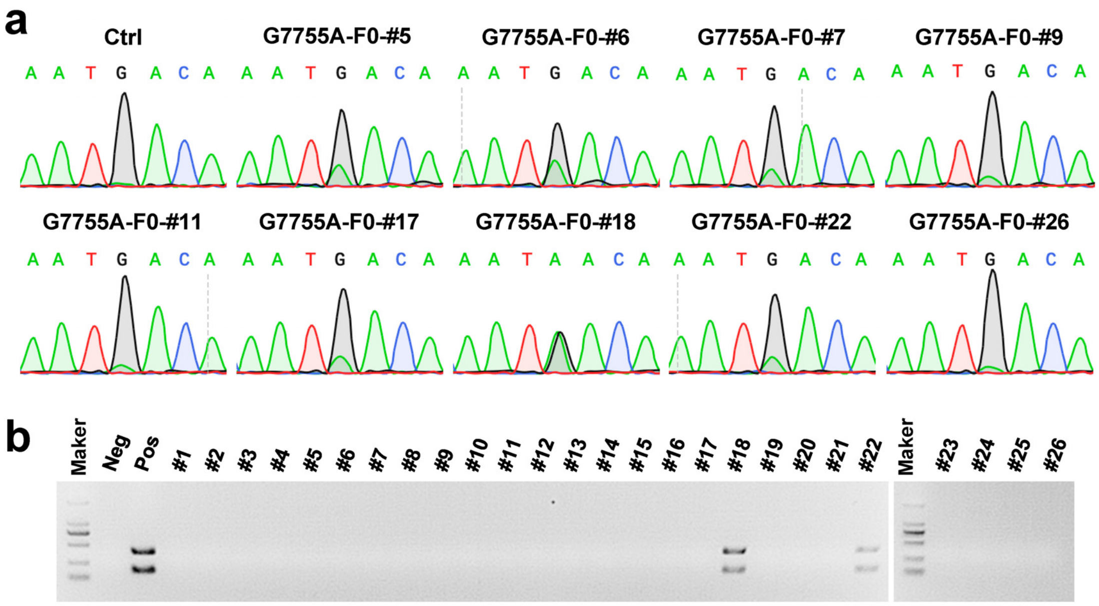

Figure S3. Mitochondrial genome editing by injection of pure DdCBE constructions.

a Sanger sequencing chromograms of G7755A founders produced by microinjection of pure DdCBE constructions.

b Detection of the integrated $DddA_{tox}$ halves in the genome of G7755A founders.

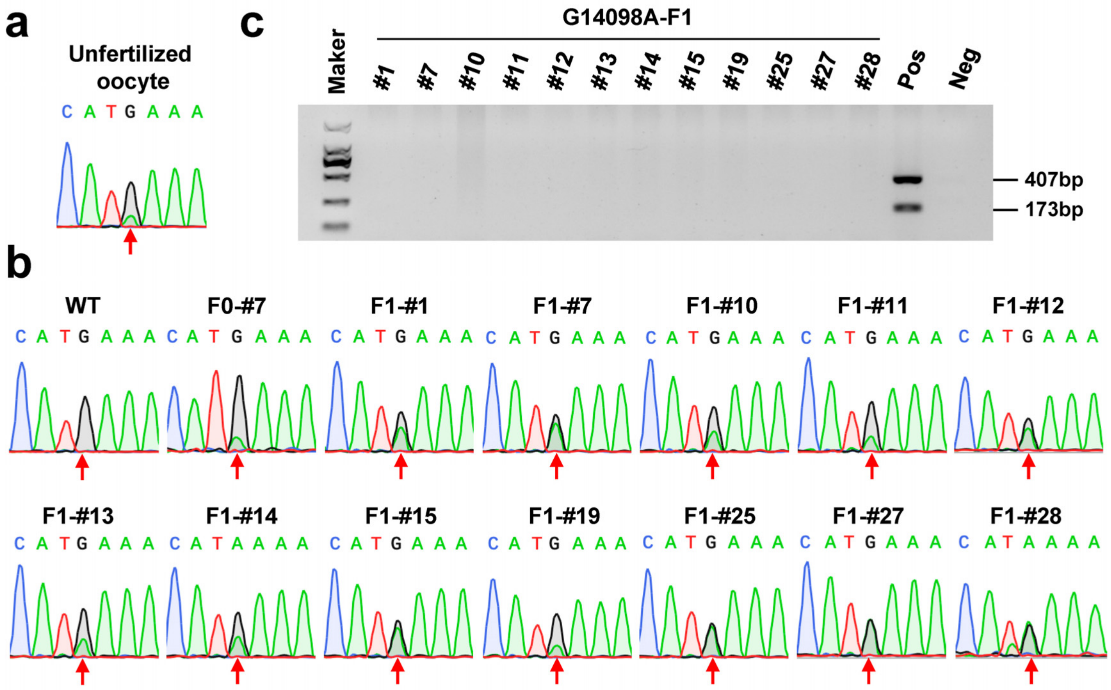

Figure S4. Germline transmission analysis of G14098A mutation.

a Representative sequence chromatograms of unfertilized oocytes from G14098A #7 founder.

b Sanger sequencing chromatograms of G14098A #7 and F1 offspring carrying G-to-A conversion at G14098. Target site is indicated by red arrow.

c Detecting the presence of DddA$_{tox}$ halves in the genome of G14098A F1 offspring.

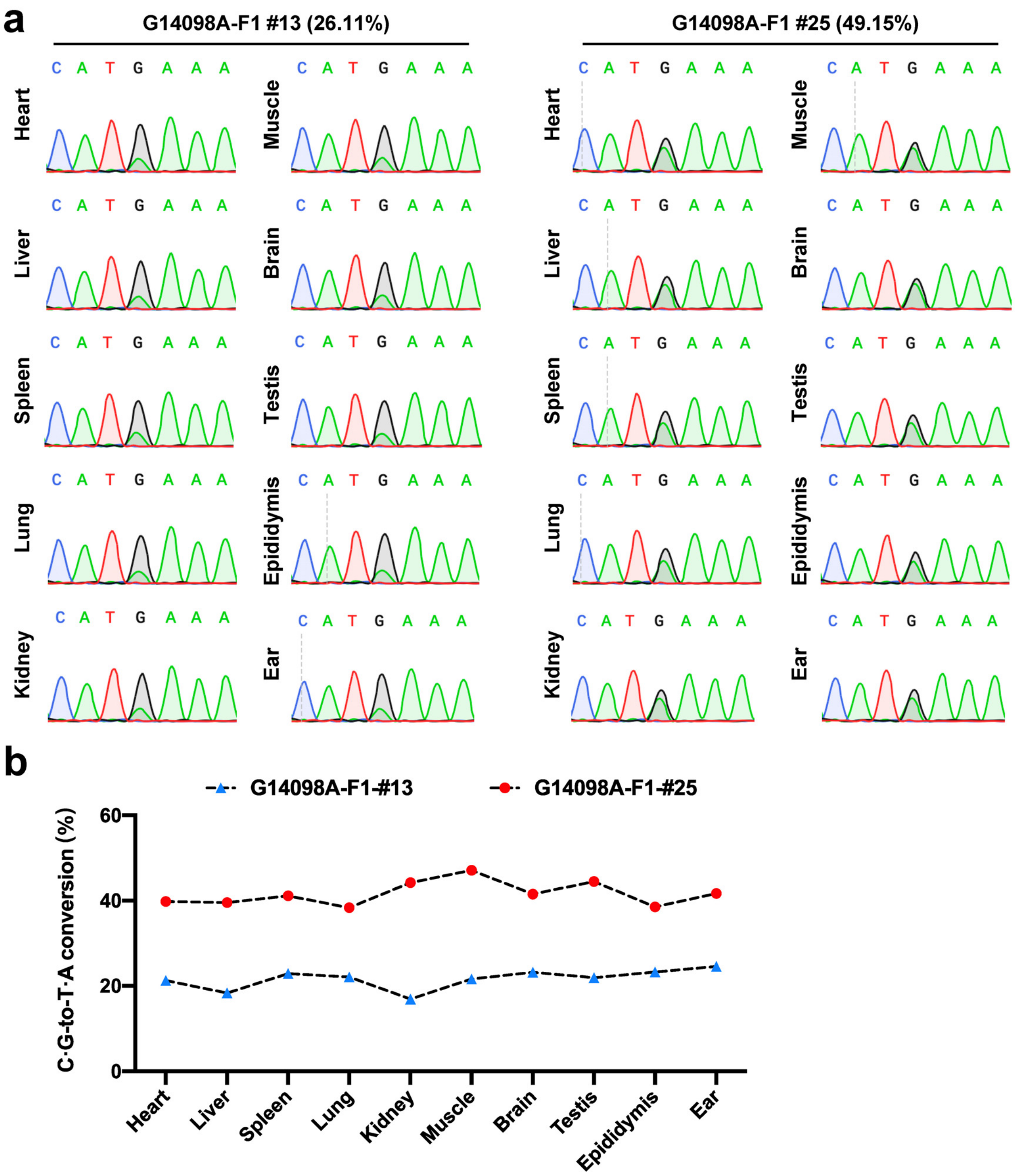

Figure S5. Mitochondrial genome editing in various tissues of G14098A F1 rat.

a Representative sequencing chromatograms of tissues from G14098A-F1 #13 and #25.
b Frequencies of G-to-A conversion at G14098 in tissues of G14098A-F1 #13 and #25.

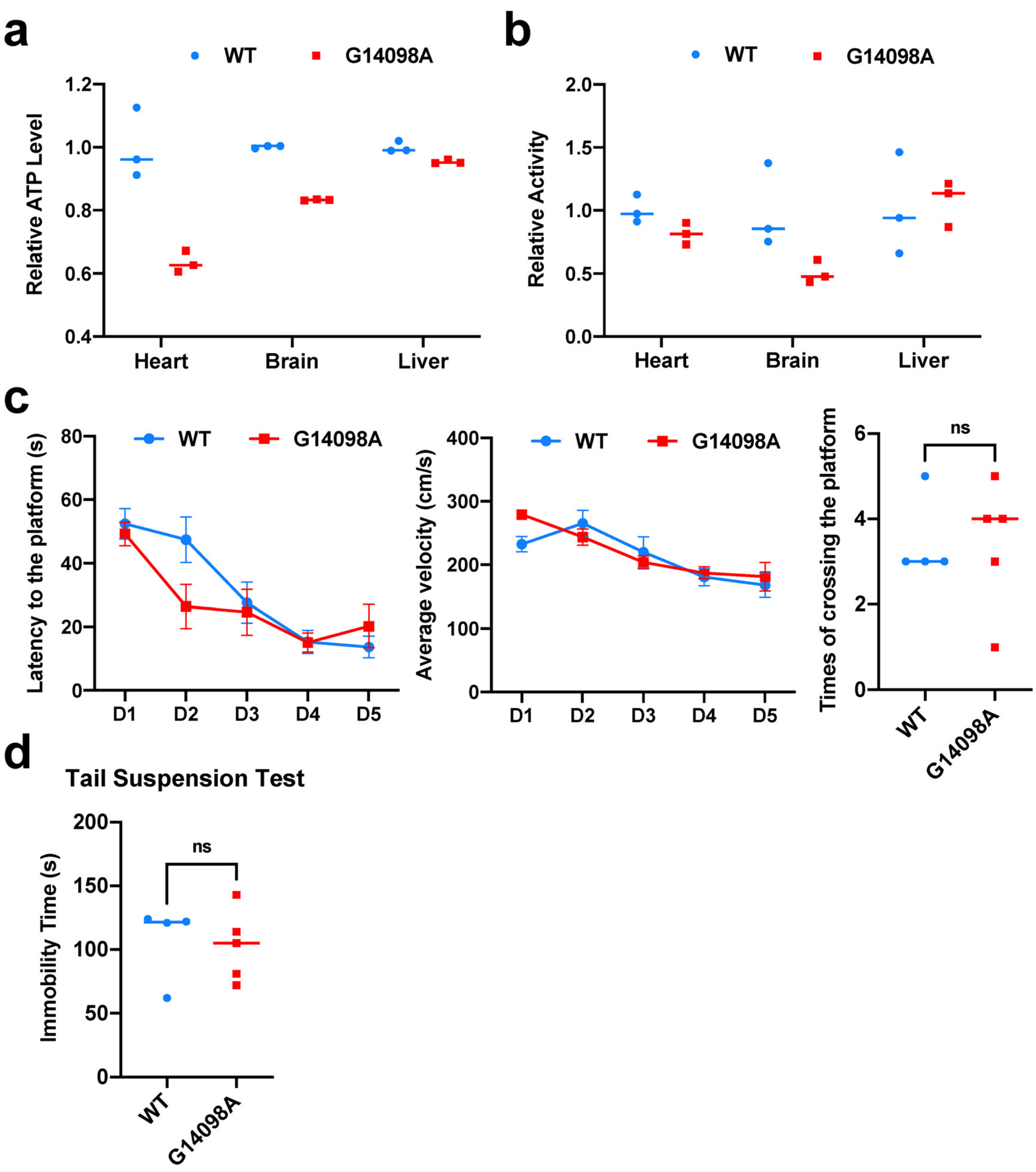

Figure S6. Analysis of ATP synthesis, complex I activity and motor ability in G14098A F1 rat.

a, **b** ATP level (**a**) and Complex I activity (**b**) in heart, brain and liver of wild-type control and G14098A founder #1. The test was repeated three times for one control rat and G14098A founder #1.

c Determination of learning ability by Morris Water Maze test.

d Determination of depression by Tail Suspension Test.

Data of **c** and **d** are presented as means ± SEM or scatter dot plot with means (n = 4 for wild-type control, n = 5 for G14098A F1 rats). Significance was calculated with unpaired two-tailed Student's t test (ns, not significant).

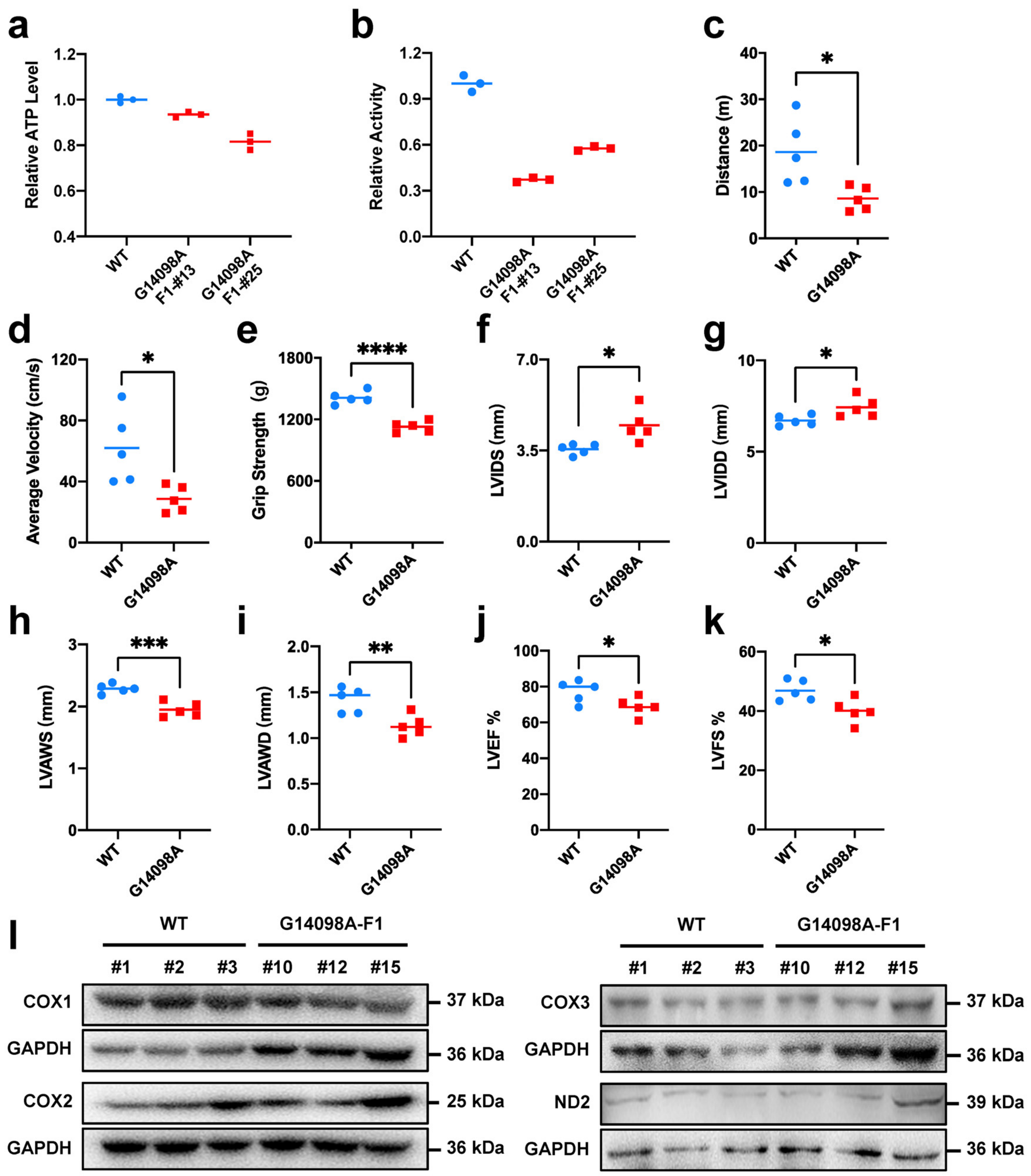

Figure S7. Phenotype analysis of G14098A F1 and F2 rat.

a,b ATP level (**a**) and Complex I activity (**b**) in heart of wild-type control and G14098A-F1 #13 and 25#. The test was repeated three times for each sample.

c,d Measuring distance (**c**) and speed (**d**) by Open Field Test of G14098A F2 rat.

e Measuring grip strength by Grip Strength Test of G14098A F2 rat.

f-k Echocardiography analysis of wild-type rats and edited F2 males. The tests include left ventricular (LV) diameter at end systole and end diastole (LVIDS, LVIDD) (**f**, **g**), LV anterior wall thickness at end systole and end diastole (LVAWS, LVAWD) (**h**, **i**), LV ejection fraction (LVEF) (**j**) and LV percent fractional shortening (LVFS) (**k**).

l Determinations of mitochondrial protein expression level in heart of wild type and G14098A-F1 rat.

Data of **c**-**k** are presented as scatter dot plot with mean (*n* = 5 for each group). Significance was calculated with unpaired two-tailed Student's t test (* *P* < 0.05, ** *P* < 0.01, *** *P* < 0.001, **** *P* < 0.0001).

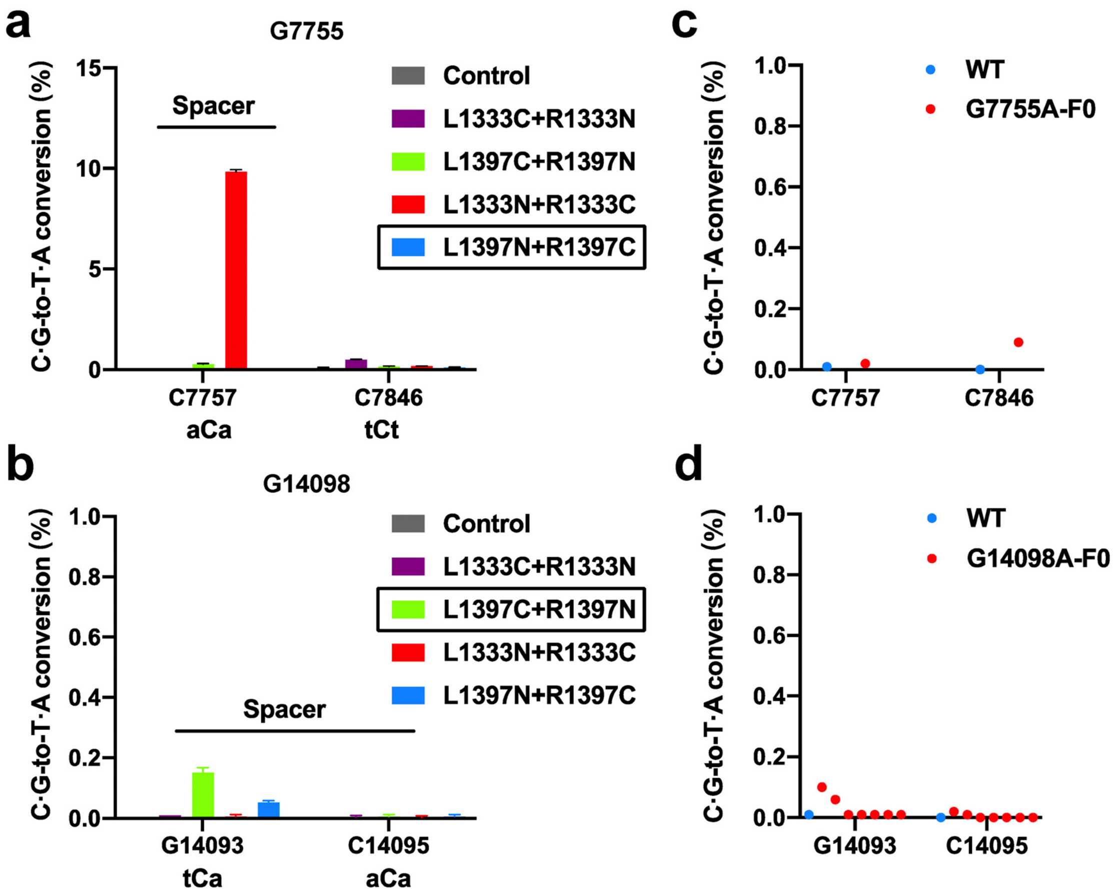

Figure S8. Deep sequencing analysis of DdCBE off-target.

a, **b** Frequencies of G7755-DdCBE (**a**) and G14098-DdCBE (**b**) mediated editing at undesired sites in C6 cells. DdCBE pairs in the black box were selected for generation of rats.

c, **d** Analysis of off-targeted editing in G7755A (**c**) and G14098A (**d**) F0 rats.

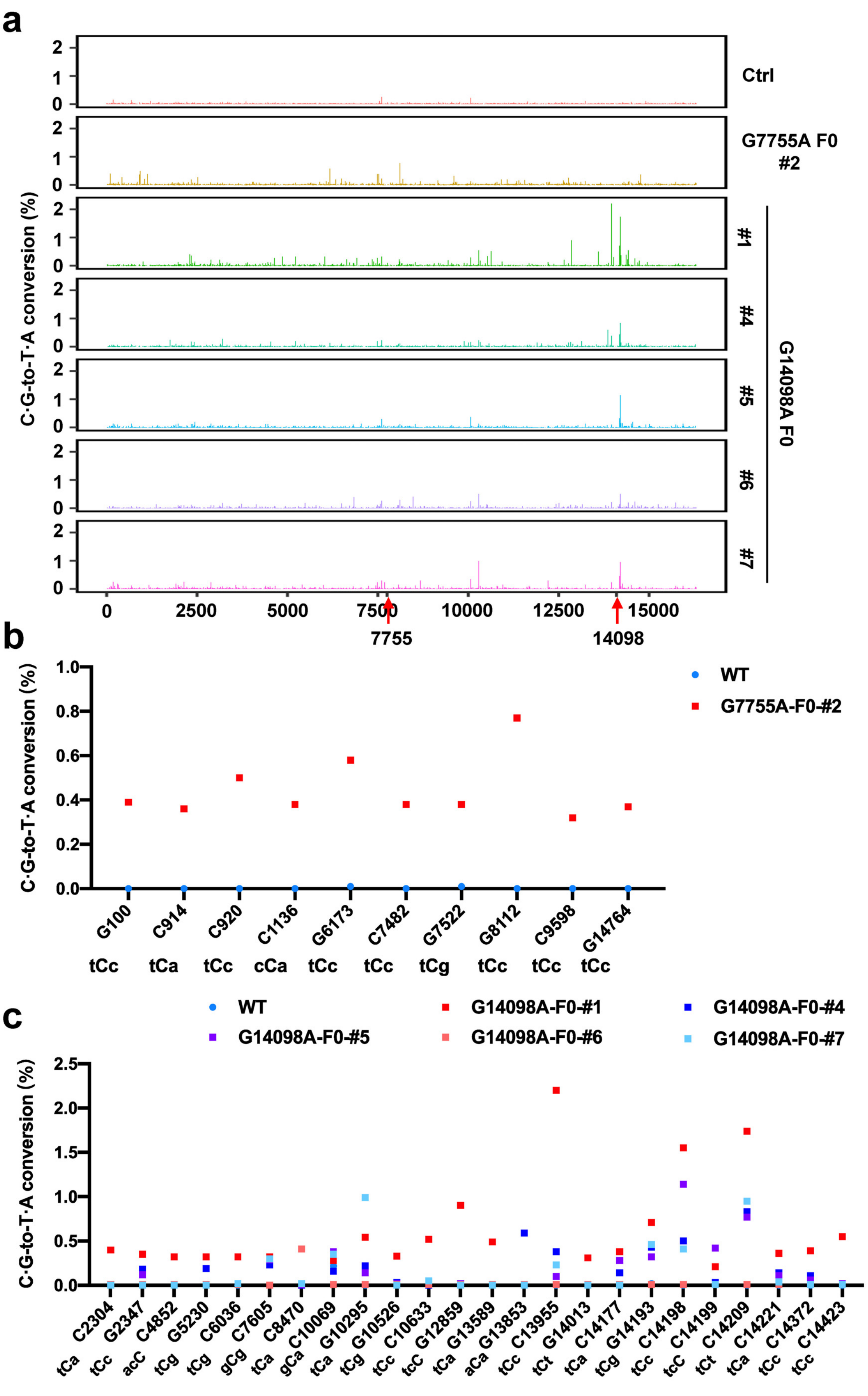

Figure S9. Off-target analysis by whole mtDNA sequencing.

a Whole mtDNA sequencing analysis of off-target sites in G7755A and G14098A founders. Wild type rat was used as control (Ctrl). Target site is indicated by red arrow.

b, **c** Off-target sites with over 0.3% editing efficiency in any sample were further analyzed in G7755A (**b**) and G14098A (**c**) founders. The motifs of off-target sites are shown in bottom.

Table S1. DdCBE injection summary.

Method	Target site	Con. (ng/μL)	No. of injected zygotes	No. of transplanted zygotes	No. of pups	No. of edited pups	Mutation load
mRNA	G7755	30	53	32	18	0	/
		100	388	212	66	0	/
		200	115	63	20	0	/
	G14098	100	62	25	9	0	/
		200	54	20	6	0	/
PB	G7755	10+30	97	24	4	1	0-36.33%
	G14098	10+30	93	78	7	7	3.9-46.73%
PB only	G7755	10	72	63	26	9	Sanger seq

Table S2. Summary of germline transmission of mtDNA mutations.

Target site	Founder rat	No. of F1	No. of edited F1 (rate)	Mutation load
G14098	#7	28	12 (42.86%)	15.92%-49.15%

Table S3. Information of edited rat.

Generation	No.	Sex	Mutation load
G7755A founder	#2	♂	36.33%
G14098A founder	#1	♂	46.73%
	#2	♀	8.30%
	#3	♂	3.91%
	#4	♂	22.99%
	#5	♂	24.92%
	#6	♂	17.04%
	#7	♀	21.73%
Offspring of G14098A founder #7	#1	♀	39.77%
	#7	♀	43.31%
	#10	♂	31.50%
	#11	♀	21.30%
	#12	♂	40.10%
	#13	♂	26.11%
	#14	♀	29.11%
	#15	♂	43.79%
	#19	♀	15.92%
	#25	♂	49.15%
	#27	♀	49.00%
	#28	♀	41.31%
Offspring of G14098A F1 females	#2	♂	39.57%
	#5	♂	36.85%
	#31	♂	42.27%
	#47	♂	31.95%
	#50	♂	43.31%

Table S4. Information of primers.

	Primers	Sequence (5'-3')
**Primers for RVD**	RVD seq Fwd	TGACCGCAGTGGAGGCAGTG
	RVD seq Rev	TGACCGCAGTGGAGGCAGTG
**Primers for genotyping**	rat-G7755-Fwd	TGCTCTGAAATTTGCGGCTC
	rat-G7755-Rev	AGTCGGTTGCTGATTAGGCG
	rat-G14098-Fwd	TCAAGTCTCCGGGTACTCCT
	rat-G14098-Rev	AAATATTGAGGCGCCGTTGG
**Primers for DddA$_{tox}$ halves**	Halves Fwd	TGCGCTGGATGCAGTGAA
	Halves Rev	CTGTCTCCTTCTCGATGATGTC
**Barcoded Primers for PCR1**	i5-rat-G7755-Fwd1	ACACTCTTTCCCTACACGACGCTCTTCCGATCTCAAA<br>ACTCATTGCGAAGCTTAGAGCG
	i5-rat-G7755-Fwd2	ACACTCTTTCCCTACACGACGCTCTTCCGATCTTCAA<br>ACTCATTGCGAAGCTTAGAGCG
	i5-rat-G7755-Fwd3	ACACTCTTTCCCTACACGACGCTCTTCCGATCTACCA<br>ACTCATTGCGAAGCTTAGAGCG
	i7-rat-G7755-Rev	GTGACTGGAGTTCAGACGTGTGCTCTTCCGATCTTGT<br>GGCTATAGTTTTGGGGGAGG
	i5-rat-G14098-Fwd1	ACACTCTTTCCCTACACGACGCTCTTCCGATCTCAAA<br>GCCCACCATAAATAGGTGAAGG
	i5-rat-G14098-Fwd2	ACACTCTTTCCCTACACGACGCTCTTCCGATCTTCAA<br>GCCCACCATAAATAGGTGAAGG
	i5-rat-G14098-Fwd3	ACACTCTTTCCCTACACGACGCTCTTCCGATCTACCA<br>GCCCACCATAAATAGGTGAAGG
	i7-rat-G14098-Rev	GTGACTGGAGTTCAGACGTGTGCTCTTCCGATCTTAG<br>GGGGTGAGATTTTCGGAT
**Primers for long-range PCR**	rat-mtDNA-F1-Fwd	ACTCGTTAGCCCAACACAGG
	rat-mtDNA-F1-Rev	AAACCTACTGCTGCTTCGCA
	rat-mtDNA-F2-Fwd	TCGCTCCCACTTAATATCTACTCT
	rat-mtDNA-F2-Rev	TTAGTCACTGGGCAGGCAATG

Supplementary sequence

Amino acids of G7755 DdCBE using for mtDNA editing are annotated as: red for MTS, italics for linker, yellow for flag tag, green for N&C-terminal domain, underlined for RVDs, purple for split DddA$_{tox}$ halves, cyan for UGI. Only RVDs sequences are showed for G14098 DdCBE.

Rat G7755A Left TALE-G1333C

MLGFVGRVAAAPASGALRRLTPSASLPPAQLLLRAAPTAVHPVRDYAAQTSESGGGGSPG*AAA*DY
KDDDDK*GS*VDLRTLGYSQQQQEKIKPKVRSTVAQHHEALVGHGFTHAHIVALSQHPAALGTVAVK
YQDMIAALPEATHEAIVGVGKQWSGARALEALLTVAGELRGPPLQLDTGQLLKIAKRGGVTAVEA
VHAWRNALTGAPLNLTPEQVVAIASNGGGKQALETVQRLLPVLCQAHGLTPEQVVAIASNIGGKQ
ALETVQRLLPVLCQAHGLTPDQVVAIASNNGGKQALETVQRLLPVLCQAHGLTPEQVVAIASNIG
GKQALETVQRLLPVLCQAHGLTPDQVVAIASNNGGKQALETVQRLLPVLCQAHGLTPEQVVAIAS
NIGGKQALETVQRLLPVLCQAHGLTPDQVVAIASHDGGKQALETVQRLLPVLCQAHGLTPAQVVA
IASNIGGKQALETVQRLLPVLCQAHGLTPDQVVAIASNIGGKQALETVQRLLPVLCQAHGLTPDQ
VVAIASHDGGKQALETVQRLLPVLCQAHGLTPAQVVAIASNIGGKQALETVQRLLPVLCQAHGLT
PDQVVAIASNIGGKQALETVQRLLPVLCQAHGLTPDQVVAIASNIGGKQALETVQRLLPVLCQAH
GLTPDQVVAIASNGGGKQALETVQRLLPVLCQAHGLTPEQVVAIASHDGGKQALETVQRLLPVLC
QAHGLTPAQVVAIASNGGGRPALESIVAQLSRPDPALAALTNDHLVALACLGGRPALDAVKKGLG
*GS*PTPYPNYANAGHVEGQSALFMRDNGISEGLVFHNNPEGTCGFCVNMTETLLPENAKMTVVPPE
GAIPVKRGATGETKVFTGNSNSPKSPTKGGC*SGGS*TNLSDIIEKETGKQLVIQESILMLPEEVEE
VIGNKPESDILVHTAYDESTDENVMLLTSDAPEYKPWALVIQDSNGENKIKML

Rat G7755A Left TALE-G1397C

MLGFVGRVAAAPASGALRRLTPSASLPPAQLLLRAAPTAVHPVRDYAAQTSESGGGGSPG*AAA*DY
KDDDDK*GS*VDLRTLGYSQQQQEKIKPKVRSTVAQHHEALVGHGFTHAHIVALSQHPAALGTVAVK
YQDMIAALPEATHEAIVGVGKQWSGARALEALLTVAGELRGPPLQLDTGQLLKIAKRGGVTAVEA
VHAWRNALTGAPLNLTPEQVVAIASNGGGKQALETVQRLLPVLCQAHGLTPEQVVAIASNIGGKQ
ALETVQRLLPVLCQAHGLTPDQVVAIASNNGGKQALETVQRLLPVLCQAHGLTPEQVVAIASNIG
GKQALETVQRLLPVLCQAHGLTPDQVVAIASNNGGKQALETVQRLLPVLCQAHGLTPEQVVAIAS
NIGGKQALETVQRLLPVLCQAHGLTPDQVVAIASHDGGKQALETVQRLLPVLCQAHGLTPAQVVA
IASNIGGKQALETVQRLLPVLCQAHGLTPDQVVAIASNIGGKQALETVQRLLPVLCQAHGLTPDQ
VVAIASHDGGKQALETVQRLLPVLCQAHGLTPAQVVAIASNIGGKQALETVQRLLPVLCQAHGLT
PDQVVAIASNIGGKQALETVQRLLPVLCQAHGLTPDQVVAIASNIGGKQALETVQRLLPVLCQAH
GLTPDQVVAIASNGGGKQALETVQRLLPVLCQAHGLTPEQVVAIASHDGGKQALETVQRLLPVLC
QAHGLTPAQVVAIASNGGGRPALESIVAQLSRPDPALAALTNDHLVALACLGGRPALDAVKKGLG
*GS*AIPVKRGATGETKVFTGNSNSPKSPTKGGC*SGGS*TNLSDIIEKETGKQLVIQESILMLPEEVE
EVIGNKPESDILVHTAYDESTDENVMLLTSDAPEYKPWALVIQDSNGENKIKML

Rat G7755A Left TALE-G1333N

MLGFVGRVAAAPASGALRRLTPSASLPPAQLLLRAAPTAVHPVRDYAAQTSESGGGGSPG*AAA*DY
KDDDDK*GS*VDLRTLGYSQQQQEKIKPKVRSTVAQHHEALVGHGFTHAHIVALSQHPAALGTVAVK

YQDMIAALPEATHEAIVGVGKQWSGARALEALLTVAGELRGPPLQLDTGQLLKIAKRGGVTAVEA
VHAWRNALTGAPLNLTPEQVVAIASNGGGKQALETVQRLLPVLCQAHGLTPEQVVAIASNIGGKQ
ALETVQRLLPVLCQAHGLTPDQVVAIASNNGGKQALETVQRLLPVLCQAHGLTPEQVVAIASNIG
GKQALETVQRLLPVLCQAHGLTPDQVVAIASNNGGKQALETVQRLLPVLCQAHGLTPEQVVAIAS
NIGGKQALETVQRLLPVLCQAHGLTPDQVVAIASHDGGKQALETVQRLLPVLCQAHGLTPAQVVA
IASNIGGKQALETVQRLLPVLCQAHGLTPDQVVAIASNIGGKQALETVQRLLPVLCQAHGLTPDQ
VVAIASHDGGKQALETVQRLLPVLCQAHGLTPAQVVAIASNIGGKQALETVQRLLPVLCQAHGLT
PDQVVAIASNIGGKQALETVQRLLPVLCQAHGLTPDQVVAIASNIGGKQALETVQRLLPVLCQAH
GLTPDQVVAIASNGGGKQALETVQRLLPVLCQAHGLTPEQVVAIASHDGGKQALETVQRLLPVLC
QAHGLTPAQVVAIASNGGGRPALESIVAQLSRPDPALAALTNDHLVALACLGGRPALDAVKKGLG
*GS*GSYALGPYQISAPQLPAYNGQTVGTFYYVNDAGGLESKVFSSGG*SGGS*TNLSDIIEKETGKQL
VIQESILMLPEEVEEVIGNKPESDILVHTAYDESTDENVMLLTSDAPEYKPWALVIQDSNGENKI
KML

Rat G7755A Left TALE-G1397N

MLGFVGRVAAAPASGALRRLTPSASLPPAQLLLRAAPTAVHPVRDYAAQTSESGGGGSPG*AAA*DY
KDDDDK*GS*VDLRTLGYSQQQQEKIKPKVRSTVAQHHEALVGHGFTHAHIVALSQHPAALGTVAVK
YQDMIAALPEATHEAIVGVGKQWSGARALEALLTVAGELRGPPLQLDTGQLLKIAKRGGVTAVEA
VHAWRNALTGAPLNLTPEQVVAIASNGGGKQALETVQRLLPVLCQAHGLTPEQVVAIASNIGGKQ
ALETVQRLLPVLCQAHGLTPDQVVAIASNNGGKQALETVQRLLPVLCQAHGLTPEQVVAIASNIG
GKQALETVQRLLPVLCQAHGLTPDQVVAIASNNGGKQALETVQRLLPVLCQAHGLTPEQVVAIAS
NIGGKQALETVQRLLPVLCQAHGLTPDQVVAIASHDGGKQALETVQRLLPVLCQAHGLTPAQVVA
IASNIGGKQALETVQRLLPVLCQAHGLTPDQVVAIASNIGGKQALETVQRLLPVLCQAHGLTPDQ
VVAIASHDGGKQALETVQRLLPVLCQAHGLTPAQVVAIASNIGGKQALETVQRLLPVLCQAHGLT
PDQVVAIASNIGGKQALETVQRLLPVLCQAHGLTPDQVVAIASNIGGKQALETVQRLLPVLCQAH
GLTPDQVVAIASNGGGKQALETVQRLLPVLCQAHGLTPEQVVAIASHDGGKQALETVQRLLPVLC
QAHGLTPAQVVAIASNGGGRPALESIVAQLSRPDPALAALTNDHLVALACLGGRPALDAVKKGLG
*GS*GSYALGPYQISAPQLPAYNGQTVGTFYYVNDAGGLESKVFSSGGPTPYPNYANAGHVEGQSAL
FMRDNGISEGLVFHNNPEGTCGFCVNMTETLLPENAKMTVVPPEG*SGGS*TNLSDIIEKETGKQLV
IQESILMLPEEVEEVIGNKPESDILVHTAYDESTDENVMLLTSDAPEYKPWALVIQDSNGENKIK
ML

Rat G7755A Right TALE-G1333C

MLGFVGRVAAAPASGALRRLTPSASLPPAQLLLRAAPTAVHPVRDYAAQTSESGGGGSPG*AAA*DY
KDDDDK*GS*VDLRTLGYSQQQQEKIKPKVRSTVAQHHEALVGHGFTHAHIVALSQHPAALGTVAVK
YQDMIAALPEATHEAIVGVGKQWSGARALEALLTVAGELRGPPLQLDTGQLLKIAKRGGVTAVEA
VHAWRNALTGAPLNLTPEQVVAIASNNGGKQALETVQRLLPVLCQAHGLTPEQVVAIASNGGGKQ
ALETVQRLLPVLCQAHGLTPEQVVAIASNNGGKQALETVQRLLPVLCQAHGLTPEQVVAIASNNG
GKQALETVQRLLPVLCQAHGLTPEQVVAIASNIGGKQALETVQRLLPVLCQAHGLTPDQVVAIAS
NGGGKQALETVQRLLPVLCQAHGLTPEQVVAIASNNGGKQALETVQRLLPVLCQAHGLTPEQVVA
IASNGGGKQALETVQRLLPVLCQAHGLTPEQVVAIASNNGGKQALETVQRLLPVLCQAHGLTPEQ
VVAIASNGGGKQALETVQRLLPVLCQAHGLTPEQVVAIASHDGGKQALETVQRLLPVLCQAHGLT
PAQVVAIASNGGGKQALETVQRLLPVLCQAHGLTPEQVVAIASNIGGKQALETVQRLLPVLCQAH
GLTPDQVVAIASNNGGKQALETVQRLLPVLCQAHGLTPEQVVAIASNGGGKQALETVQRLLPVLC

QAHGLTPEQVVAIASNGGGRPALESIVAQLSRPDPALAALTNDHLVALACLGGRPALDAVKKGLG
*GS*PTPYPNYANAGHVEGQSALFMRDNGISEGLVFHNNPEGTCGFCVNMTETLLPENAKMTVVPPE
GAIPVKRGATGETKVFTGNSNSPKSPTKGGC*SGGS*TNLSDIIEKETGKQLVIQESILMLPEEVEE
VIGNKPESDILVHTAYDESTDENVMLLTSDAPEYKPWALVIQDSNGENKIKML

Rat G7755A Right TALE-G1397C

MLGFVGRVAAAPASGALRRLTPSASLPPAQLLLRAAPTAVHPVRDYAAQTSESGGGGSPG*AAA*DY
KDDDDK*GS*VDLRTLGYSQQQQEKIKPKVRSTVAQHHEALVGHGFTHAHIVALSQHPAALGTVAVK
YQDMIAALPEATHEAIVGVGKQWSGARALEALLTVAGELRGPPLQLDTGQLLKIAKRGGVTAVEA
VHAWRNALTGAPLNLTPEQVVAIASNNGGKQALETVQRLLPVLCQAHGLTPEQVVAIASNGGGKQ
ALETVQRLLPVLCQAHGLTPEQVVAIASNNGGKQALETVQRLLPVLCQAHGLTPEQVVAIASNNG
GKQALETVQRLLPVLCQAHGLTPEQVVAIASNIGGKQALETVQRLLPVLCQAHGLTPDQVVAIAS
NGGGKQALETVQRLLPVLCQAHGLTPEQVVAIASNNGGKQALETVQRLLPVLCQAHGLTPEQVVA
IASNGGGKQALETVQRLLPVLCQAHGLTPEQVVAIASNNGGKQALETVQRLLPVLCQAHGLTPEQ
VVAIASNGGGKQALETVQRLLPVLCQAHGLTPEQVVAIASHDGGKQALETVQRLLPVLCQAHGLT
PAQVVAIASNGGGKQALETVQRLLPVLCQAHGLTPEQVVAIASNIGGKQALETVQRLLPVLCQAH
GLTPDQVVAIASNNGGKQALETVQRLLPVLCQAHGLTPEQVVAIASNGGGKQALETVQRLLPVLC
QAHGLTPEQVVAIASNGGGRPALESIVAQLSRPDPALAALTNDHLVALACLGGRPALDAVKKGLG
*GS*AIPVKRGATGETKVFTGNSNSPKSPTKGGC*SGGS*TNLSDIIEKETGKQLVIQESILMLPEEVE
EVIGNKPESDILVHTAYDESTDENVMLLTSDAPEYKPWALVIQDSNGENKIKML

Rat G7755A Right TALE-G1333N

MLGFVGRVAAAPASGALRRLTPSASLPPAQLLLRAAPTAVHPVRDYAAQTSESGGGGSPG*AAA*DY
KDDDDK*GS*VDLRTLGYSQQQQEKIKPKVRSTVAQHHEALVGHGFTHAHIVALSQHPAALGTVAVK
YQDMIAALPEATHEAIVGVGKQWSGARALEALLTVAGELRGPPLQLDTGQLLKIAKRGGVTAVEA
VHAWRNALTGAPLNLTPEQVVAIASNNGGKQALETVQRLLPVLCQAHGLTPEQVVAIASNGGGKQ
ALETVQRLLPVLCQAHGLTPEQVVAIASNNGGKQALETVQRLLPVLCQAHGLTPEQVVAIASNNG
GKQALETVQRLLPVLCQAHGLTPEQVVAIASNIGGKQALETVQRLLPVLCQAHGLTPDQVVAIAS
NGGGKQALETVQRLLPVLCQAHGLTPEQVVAIASNNGGKQALETVQRLLPVLCQAHGLTPEQVVA
IASNGGGKQALETVQRLLPVLCQAHGLTPEQVVAIASNNGGKQALETVQRLLPVLCQAHGLTPEQ
VVAIASNGGGKQALETVQRLLPVLCQAHGLTPEQVVAIASHDGGKQALETVQRLLPVLCQAHGLT
PAQVVAIASNGGGKQALETVQRLLPVLCQAHGLTPEQVVAIASNIGGKQALETVQRLLPVLCQAH
GLTPDQVVAIASNNGGKQALETVQRLLPVLCQAHGLTPEQVVAIASNGGGKQALETVQRLLPVLC
QAHGLTPEQVVAIASNGGGRPALESIVAQLSRPDPALAALTNDHLVALACLGGRPALDAVKKGLG
*GS*GSYALGPYQISAPQLPAYNGQTVGTFYYVNDAGGLESKVFSSGG*SGGS*TNLSDIIEKETGKQL
VIQESILMLPEEVEEVIGNKPESDILVHTAYDESTDENVMLLTSDAPEYKPWALVIQDSNGENKI
KML

Rat G7755A Right TALE-G1397N

MLGFVGRVAAAPASGALRRLTPSASLPPAQLLLRAAPTAVHPVRDYAAQTSESGGGGSPG*AAA*DY
KDDDDK*GS*VDLRTLGYSQQQQEKIKPKVRSTVAQHHEALVGHGFTHAHIVALSQHPAALGTVAVK
YQDMIAALPEATHEAIVGVGKQWSGARALEALLTVAGELRGPPLQLDTGQLLKIAKRGGVTAVEA
VHAWRNALTGAPLNLTPEQVVAIASNNGGKQALETVQRLLPVLCQAHGLTPEQVVAIASNGGGKQ
ALETVQRLLPVLCQAHGLTPEQVVAIASNNGGKQALETVQRLLPVLCQAHGLTPEQVVAIASNNG

GKQALETVQRLLPVLCQAHGLTPEQVVAIASNIGGKQALETVQRLLPVLCQAHGLTPDQVVAIAS NGGGKQALETVQRLLPVLCQAHGLTPEQVVAIASNNGGKQALETVQRLLPVLCQAHGLTPEQVVA IASNGGGKQALETVQRLLPVLCQAHGLTPEQVVAIASNNGGKQALETVQRLLPVLCQAHGLTPEQ VVAIASNGGGKQALETVQRLLPVLCQAHGLTPEQVVAIASHDGGKQALETVQRLLPVLCQAHGLT PAQVVAIASNGGGKQALETVQRLLPVLCQAHGLTPEQVVAIASNIGGKQALETVQRLLPVLCQAH GLTPDQVVAIASNNGGKQALETVQRLLPVLCQAHGLTPEQVVAIASNGGGKQALETVQRLLPVLC QAHGLTPEQVVAIASNGGGRPALESIVAQLSRPDPALAALTNDHLVALACLGGRPALDAVKKGLG *GS*GSYALGPYQISAPQLPAYNGQTVGTFYYVNDAGGLESKVFSSGGPTPYPNYANAGHVEGQSAL FMRDNGISEGLVFHNNPEGTCGFCVNMTETLLPENAKMTVVPPEG*SGGS*TNLSDIIEKETGKQLV IQESILMLPEEVEEVIGNKPESDILVHTAYDESTDENVMLLTSDAPEYKPWALVIQDSNGENKIK ML

Rat G14098A Left TALE

LTPEQVVAIASNGGGKQALETVQRLLPVLCQAHGLTPEQVVAIASNGGGKQALETVQRLLPVLCQ AHGLTPEQVVAIASNIGGKQALETVQRLLPVLCQAHGLTPDQVVAIASNIGGKQALETVQRLLPV LCQAHGLTPDQVVAIASHDGGKQALETVQRLLPVLCQAHGLTPAQVVAIASNGGGKQALETVQRL LPVLCQAHGLTPEQVVAIASNNGGKQALETVQRLLPVLCQAHGLTPEQVVAIASNGGGKQALETV QRLLPVLCQAHGLTPEQVVAIASNNGGKQALETVQRLLPVLCQAHGLTPEQVVAIASNIGGKQAL ETVQRLLPVLCQAHGLTPDQVVAIASHDGGKQALETVQRLLPVLCQAHGLTPAQVVAIASNGGGK QALETVQRLLPVLCQAHGLTPEQVVAIASNIGGKQALETVQRLLPVLCQAHGLTPDQVVAIASNI GGKQALETVQRLLPVLCQAHGLTPDQVVAIASNGGGRPALE

Rat G14098A Right TALE

LTPEQVVAIASNIGGKQALETVQRLLPVLCQAHGLTPDQVVAIASNNGGKQALETVQRLLPVLCQ AHGLTPEQVVAIASNGGGKQALETVQRLLPVLCQAHGLTPEQVVAIASNGGGKQALETVQRLLPV LCQAHGLTPEQVVAIASNNGGKQALETVQRLLPVLCQAHGLTPEQVVAIASNIGGKQALETVQRL LPVLCQAHGLTPDQVVAIASNIGGKQALETVQRLLPVLCQAHGLTPDQVVAIASNGGGKQALETV QRLLPVLCQAHGLTPEQVVAIASNGGGKQALETVQRLLPVLCQAHGLTPEQVVAIASNIGGKQAL ETVQRLLPVLCQAHGLTPDQVVAIASHDGGKQALETVQRLLPVLCQAHGLTPAQVVAIASNIGGK QALETVQRLLPVLCQAHGLTPDQVVAIASNIGGKQALETVQRLLPVLCQAHGLTPDQVVAIASHD GGKQALETVQRLLPVLCQAHGLTPAQVVAIASNNGGKQALETVQRLLPVLCQAHGLTPEQVVAIA SNIGGKQALETVQRLLPVLCQAHGLTPDQVVAIASNGGGRPALE
